# Global cardiometabolic alliance: A clinical and research initiative addressing the 21st century pandemic of insulin resistance‐associated disease

**DOI:** 10.1111/dom.70033

**Published:** 2025-08-14

**Authors:** Robert H. Eckel, Andrew J. Krentz, Stephan Jacob, Peter Libby

**Affiliations:** ^1^ Division of Endocrinology Metabolism and Diabetes at the University of Colorado Anschutz Medical Campus Aurora Colorado USA; ^2^ School of Life Course & Population Sciences, King's College London London UK; ^3^ Kardio‐Metabolisches Institut Villingen Germany; ^4^ Brigham and Women's Hospital Boston Massachusetts USA

**Keywords:** cardiovascular disease, diabetes complications, insulin resistance, obesity care

## 1 THE PROBLEM AND GOALS

The pandemic of obesity is upon us, and the future is not encouraging. Using the new definition of clinical obesity,^1^ where excessive body adipose tissue is associated with medical illnesses, the prevalence of clinical obesity in the US, UK, China and Spain approaches 70% (under review). Most of these clinical illnesses reside within the expanding definition of the metabolic syndrome, which is a cluster that relates obesity with cardiovascular disease and diabetes in an elementary manner using five metabolic criteria.^2^

The term cardiometabolic medicine began to be used with the increasing incidence and prevalence of obesity in the late twentieth century. In a Call to Action by the American Heart Association (AHA) in 1998, for the first time obesity was labelled as a major risk factor for coronary heart disease (CHD).^3^ Months later the World Health Organization (WHO) first established a definition of metabolic syndrome using criteria that were impractical to apply in clinical practice, namely, measuring insulin resistance under hyperinsulinaemic euglycaemic conditions.^4^ The clinical importance of metabolic syndrome was to embrace a focus on managing CVD risk more appropriate for the current era extending beyond reducing levels of single contributors LDL cholesterol (LDL‐C) and the other traditional Framingham factors. Among the most cited is the Harmonized definition established by the National Heart Lung Blood Institute (NHLBI), AHA, International Diabetes Foundation (IDF), International Atherosclerosis Society (IAS) and World Heart Federation (WHF) which defined the syndrome by 3 of the following 5 criteria: abdominal circumference – men >85–102 cm and women >80–97 cm (adjusted locally around the world), triglycerides >150 mg/dL (1.7 mmol/L), high‐density lipoprotein (HDL)‐cholesterol – men <40 mg/dL (1.03 mmol/L), women <50 mg/dL (1.29 mmol/L), blood pressure > 130/85 or on blood pressure lowering medications and fasting glucose >100 mg/dL (5.5 mmol/L).^5^

Despite the value of metabolic syndrome concept, the ability to predict CVD may simply reflect the sum of the components.^6^ Moreover, to be addressed in the next section, the current definitions fall far short of defining risk for and mechanisms of CVD and other aspects of the expanding impact of cardiometabolic disease. Presently, obesity is a pandemic, and according to the World Obesity Atlas 2025 report, by 2030, 50% of adult men and women will be living with a body mass index ≥30 mg/kg^2^ (https://data.worldobesity.org/publications/world-obesity-atlas-2025-v7.pdf) with certain increases in the incidence of all other outcomes that relate to the newly established criteria for clinical obesity.^1^

Of interest and importance, according to the Center for Disease Control National Vital Statistics System for the USA, 2023, **~**1 050 000 or 50% of deaths were from heart disease, stroke, diabetes, kidney disease, chronic liver disease and cirrhosis (Products‐Data Briefs‐Number 521‐December 2024). The future is not bright with increasing numbers of individuals becoming obese in childhood and adolescence, a tide that is difficult to overturn in adults. To address this global public health threat of obesity, metabolic syndrome and the many other related cardiometabolic risks, we have established the Global Cardiometabolic Alliance. **We aim to stimulate the interest of clinicians and clinical researchers across a range of medical specialties, and public health officials who share the vision of better care for people living with cardiometabolic diseases to become more informed and proactive on addressing this major systemic health challenge of the twenty‐first century**.

## 2 MECHANISMS OF CARDIOMETABOLIC DISEASE

A brief history may be relevant. In the 1920s, Kylin, a Swedish physician, described an association between hypertension, hyperglycaemia and gout.^7^ Then, in 1947, Vague drew attention to upper body adiposity as the obesity phenotype commonly seen with metabolic abnormalities associated with type 2 diabetes and CVD.^8^ Subsequently, considerable evidence has accumulated that insulin resistance characterises the metabolic syndrome cluster.^9^ Excessive release of non‐esterified'fre' fatty acids, importantly from visceral or intra‐abdominal adipose tissue, results in hepatic, skeletal and cardiac muscle, and vascular wall pathophysiologic defects that contribute to the elements of the current definitions.^10^ However, metabolic syndrome and associated insulin resistance stretch beyond these definitions and include inflammation, metabolic dysfunction‐associated steatotic liver disease, polycystic ovarian syndrome, hypertensive and metabolic complications of pregnancy, hyperuricaemia and gout, renal disease, cigarette smoking, sedentary behaviour, increased apolipoprotein (apo)B, small dense and biochemically altered low‐density lipoprotein (LDL) and HDL particles, increased apolipoprotein CIII, increased fibrinogen, decreased fibrinolysis, microalbuminuria, reduced asymmetric dimethylarginine and even neurodegenerative diseases.^11^, ^12^ Thus, insulin resistance results in metabolic, cardiovascular, renal, hepatic and CNS diseases that we could call cardio‐renal‐hepatic‐metabolic‐neurodegenerative medicine or more simply and holistically, cardiometabolic medicine.

## 3 RECENT ADVANCEMENTS IN CARE FOR CARDIOMETABOLIC DISORDERS

Extensive clinical trial evidence supports the use of newer pharmacotherapies, notably glucagon‐like peptide (GLP)‐1 receptor agonists and sodium‐glucose cotransporter (SGLT)‐2 inhibitors to improve cardio–renal–vascular outcomes.^13^, ^14^, ^15^

Exploiting the changing opportunities for improved care for cardio‐renal‐metabolic disease requires a coordinated multidisciplinary approach.

## 4 UNMET CLINICAL NEEDS

New structures for education and clinical workup that recognise stages of disease are needed^16^, ^17^ (Table 1). Delivery of improved clinical care for cardiometabolic disease could be enhanced by structured pathways for detection and staging.^18^ To enhance practical utility, this effort could comprise simple, easy, and inexpensive clinical, laboratory and imaging tests ranging from a minimum set to more sophisticated optional investigations according to available health care resources.

**TABLE 1:** Deficiencies in cardiometabolic disease management.

Incomplete risk factor detection and disease staging^a^ Insufficient emphasis on excess and/or dysfunctional adiposity as fundamental disease driver Suboptimal use of GLP‐1 RAs, SGLT‐2 inhibitors and other novel pharmacotherapies according to rapidly accumulating clinical trial evidence^b^ Deficiencies in education and support for healthcare professionals, patients and their families. Structured cardiometabolic care not established in most clinics Barriers to accessibility of many current therapies in a widespread and equitable manner

^a^ Med Internet Res. 2020 Dec 7;22(12):e18725. 10.2196/18725.

^b^ Cardiovasc Drugs Ther 2022 Nov 15;38(2):347–357. 10.1007/s10557‐022‐07403‐2.

## 5 ROLES OF PHYSICIAN TRAINING AND SERVICE REDESIGN IN IMPROVING THE PREVENTION AND MANAGEMENT OF CARDIOMETABOLIC DISORDERS

Following definitions of metabolic syndrome, multiple meetings followed beginning with the Cardiometabolic Health Congress (CMHC) in Boston in 2005, now in its 20th year. CMHC recently developed a Certificate Course to become a Certified Cardiometabolic Health Professional (CCHP), designed for any health care professional seeking to learn the fundamentals of the intersections of cardiometabolic risk factors, and the core principles in the management of all aspects of cardiometabolic medicine (CMHC Foundations of Cardiometabolic Health Certification Course | Cardiometabolic Health Congress). Upon completion of all focus areas, learners receive a certificate acknowledging their successful completion of the 75+ hour course. This course is appropriate for clinicians and allied health professionals across a full spectrum of clinical experience including physicians, physician assistants, nurse practitioners, registered nurses, pharmacists, diabetologists and dietitians who are in any phase of training, or even experienced physicians who seek a deeper knowledge and competence in cardiometabolic health.

Other options could be 1‐year fellowships directed to one aspect of cardiometabolic medicine, namely diabetes, obesity, or chronic kidney disease; a 1‐year fellowship in cardiometabolic medicine which would require an already established curriculum at an institution; or optimally, a new medical subspeciality with in‐depth training over a 2–3‐year period.^19^ This programme would be inclusive and cover all the components of cardiometabolic medicine without many aspects of classic subspecialty training, namely, coronary intervention, electrophysiology, advanced heart failure, thyroid disease, metabolic bone disease, reproductive endocrinology, hypothalamic/pituitary/adrenal disease, renal biopsy, haemodialysis, peritoneal dialysis, renal transplantation, liver biopsy or cirrhosis management. An example of the proposed training programme as modified is shown in Table 2.^20^

**TABLE 2:** Proposal for a postgraduate training programme in cardiometabolic disease.

Year 1	Year 2	Year 3
Clinics	Clinics	Clinics
Diabetes/Obesity/Lipid –12 mo General Cardiology – 12 mo Nephrology – 6 mo Hepatology – 2 mo Neurology – 2 mo	Diabetes/Obesity/Lipid – 12 mo General Cardiology – 12 mo Nephrology – 2 mo Hepatology – 2 mo Neurology – 2 mo	Diabetes/Obesity/Lipid – 6 mo General Cardiology – 6 mo Nephrology – 2 mo

Inpatient	Inpatient	Inpatient
Cardiology – 2 mo Nephrology – 2 mo Glucose management – 1 mo	Cardiology – 2 mo Nephrology – 2 mo Glucose management – 1 mo	Cardiology – 1 mo Nephrology – 1 mo Glucose management – 1 mo

Other	Other	Other
Cardiac imaging – 2 mo Cardiac rehab – 2 mo Stress testing – 2 mo Nutrition/Exercise – 2 mo Sleep medicine – 1 mo	Cardiac imaging – 3 mo Cardiac rehab – 1 mo Smoking cessation – 1 mo Leadership training – 1 mo	Electives

*Note*: See main text for details. [Correction added on 1 September 2025, after first online publication: Table 2 has been added in this version]

**An important issue to consider is what type of specialist or academician would be sufficiently proficient and responsible for teaching such complex and multidisciplinary medical issues? At present, while options for additional training are being implemented, those who educate trainees would be various specialists who have a passion, academic and clinical experience in cardiometabolic medicine. A historic comparison is geriatric medicine where training programmes began with internists and internal medicine specialists who had an interest in normal ageing and diseases more common in the elderly**.

Collaborative cross‐specialty studies, including clinical audits of real‐world practice and clinical trials, require familiarity of investigators with relevant research methodologies.

One benefit of such a programme would be reducing or eliminating the cardiometabolic shuffle, that is, between the primary care physician, cardiologist, endocrinologist, nephrologist, hepatologist and possibly the neurologist.

Optimal training would be in a clinic already established to deal with this broad spectrum of patients with multiple manifestations of cardiometabolic disease. The programme established by Mikhail Kosiborod at St. Luke's Hospital System in Kansas City^21^ provides a model. The St. Luke's programme addresses cardiometabolic disease in a holistic manner, providing team‐based, comprehensive care specifically tailored to each patient. Members from across the country and now internationally adopt the Alliance's care pathways and guideline‐based protocols, and also contribute to the Cardiometabolic Centre Alliance's National Patient Registry to measure the impact of the care delivered using this approach. Data from the Registry have been presented and are under peer review to reflect that the Alliance‐directed implementation of coordinated team‐based, comprehensive cardiometabolic risk reduction programmes produce rapid and statistically significant improvements in guideline‐directed medication therapy adoption and superior control of risk factors across multiple member sites and attenuated care disparities.

## 6 CONCLUSIONS

1. The dire need to prevent and treat the Cardio‐Renal‐Hepatic‐Metabolic‐Central Nervous System (CNS) disease pandemic while providing cost‐effective, high‐quality health care presents one of the most important health challenges of the twenty‐first century.
2. A less siloed approach would enhance prevention, treatment and management of risk factors of Cardio‐Renal‐Hepatic‐Metabolic‐CNS disease, with specialists working collaboratively with other HCPs in interdisciplinary teams.
3. Introducing Cardio‐Renal‐Hepatic‐Metabolic‐CNS Medicine clinics and a separate specialty with a comprehensive training programme introduced in medical school and extending through specialisation could prove an effective and long‐lasting approach to tackling this enormous task.
4. Until then, Cardio‐Renal‐Hepatic‐Metabolic‐CNS Medicine certificates or 1‐year fellowships could help close this gap.

**We offer these proposals in the hope that they may provide a theoretical and practical basis for improving care and prevention of these prevalent insulin resistance‐associated disorders. Moreover, we hope these proposals can be adopted to greater or lesser degrees by health care systems as resources permit**.

## FUNDING INFORMATION

There was no funding for this research.

## CONFLICT OF INTEREST STATEMENT

The authors declare no conflicts of interest.

## PEER REVIEW

The peer review history for this article is available at https://www.webofscience.com/api/gateway/wos/peer‐review/10.1111/dom.70033.

## Data Availability

N/A.
